# An evaluation of symptom domains in the 2 years before pregnancy as predictors of relapse in the perinatal period in women with severe mental illness

**DOI:** 10.1192/j.eurpsy.2021.18

**Published:** 2021-03-19

**Authors:** Sharvari Khapre, Robert Stewart, Clare Taylor

**Affiliations:** 1 Department of Psychological Medicine, Institute of Psychiatry, Psychology and Neuroscience, King’s College London, De Crespigny Park, London SE5 8AF, United Kingdom; 2 South London and Maudsley NHS Foundation Trust, London, United Kingdom; 3 Women’s College Hospital, 76 Grenville Street, Toronto, Ontario M5S 1B2, Canada

**Keywords:** cohort, perinatal, relapse, severe mental illness, symptoms

## Abstract

**Background:**

Symptoms may be more useful prognostic markers for mental illness than diagnoses. We sought to investigate symptom domains in women with pre-existing severe mental illness (SMI; psychotic and bipolar disorder) as predictors of relapse risk during the perinatal period.

**Methods:**

Data were obtained from electronic health records of 399 pregnant women with SMI diagnoses from a large south London mental healthcare provider. Symptoms within six domains characteristically associated with SMI (positive, negative, disorganization, mania, depression, and catatonia) recorded in clinical notes 2 years before pregnancy were identified with natural language processing algorithms to extract data from text, and associations investigated with hospitalization during pregnancy and 3 months postpartum.

**Results:**

Seventy-six women (19%) relapsed during pregnancy and 107 (27%) relapsed postpartum. After adjusting for covariates, disorganization symptoms showed a positive association at borderline significance with relapse during pregnancy (adjusted odds ratio [aOR] = 1.36; 95% confidence interval [CI] = 0.99–1.87 per unit increase in number of symptoms) and depressive symptoms negatively with relapse postpartum (0.78; 0.62–0.98). Restricting the sample to women with at least one recorded symptom in any given domain, higher disorganization (1.84; 1.22–2.76), positive (1.50; 1.07–2.11), and manic (1.48; 1.03–2.11) symptoms were associated with relapse during pregnancy, and disorganization (1.54; 1.08–2.20) symptom domains were associated with relapse postpartum.

**Conclusions:**

Positive, disorganization, and manic symptoms recorded in the 2 years before pregnancy were associated with increased risk of relapse during pregnancy and postpartum. The characterization of routine health records from text fields is relatively transferrable and could help inform predictive risk modelling.

## Introduction

Severe mental illness (SMI) includes psychotic and bipolar disorders. Research into relapse of SMI perinatally has primarily focused on bipolar disorder and/or psychosis postpartum, with risk estimates ranging from 17 to 47% [1–4]. In pregnancy, incidence ranges from 9 to 23% in clinical populations [1,5] and in women with schizophrenia and other nonaffective psychoses, health administrative, and clinical data estimate relapse at 13–39% in pregnancy [3,6,7] and 31% postpartum in the first 3 months [8].

The consequences of a severe perinatal relapse are potentially devastating and include child custody loss and in extreme but rare cases, suicide, or infanticide [9–11]. The UK and Australian Confidential Enquiries highlight psychiatric illness as a leading contributor to maternal mortality [9,12]. Risk factors for perinatal relapse of SMI include nonwhite ethnicity, lower income, having fewer children, being unmarried or not living with the father of the child, and an unplanned pregnancy [5,7,13–15]. Characteristics of the psychiatric disorder have also been highlighted including family history, younger onset, previous perinatal episodes, increased recurrences, recent psychiatric admissions, smoking, harmful alcohol or substance use, and psychiatric comorbidity [3,5–8,14,16,17].

The nature of the pre-existing clinical syndrome has received little attention and diagnostic categories may be limited, having overlapping genetic, neurobiological, and clinical features, limited prognostic significance alone, instability over time and co-aggregation in familial studies [18–21]. Symptoms commonly associated with SMI include positive and negative symptoms classically described in schizophrenia as well as symptoms of disorganization and catatonia [22]. However, these may also occur in affective disorders and affective symptoms such as depression and mania occur in patients with schizophrenia. There is thus a rationale for considering symptoms rather than diagnostic categories as outcome predictors. Increasing accessibility of digitized text from electronic health records has enabled, for example, extracted negative symptoms in schizophrenia to be investigated in relation to hospitalizations [23]. Our aim was to investigate the extent to which particular symptom domains in women with SMI were associated with relapse during the perinatal period.

## Methods

### Data source

Data were gathered from the South London and Maudsley Biomedical Research Centre (SLaM BRC) Clinical Record Interactive Search (CRIS). SLaM is a mental health care provider serving a south London catchment with electronic healthcare records since 2006 [24]. CRIS permits generation of de-identified research databases from within an approved governance framework (Oxfordshire Research Ethics Committee C; reference 18/SC/0372). Since its introduction, CRIS has been enhanced through external data linkages and natural language processing (NLP) [25,26]. Linkages include Hospital Episode Statistics (HES), which provide statistical data on all NHS hospital care in England, including maternity data [27]. A range of NLP algorithms for CRIS have been developed using General Architecture for Text Engineering software [28] which extracts information from text, accounting for the linguistic context of words and phrases. Derived and validated algorithms developed include those extracting information on diagnoses, medication, and a range of psychiatric symptoms [25,29].

### Cohort assembly

This study made use of a previously characterized retrospective cohort of pregnant women with preexisting SMI, assembled using CRIS and HES [30]. The cohort comprised all recorded women with preexisting SMI who had been pregnant between 2007 and 2011 The linkage with HES was used to identify instances of pregnancy within the study period. Delivery episodes were identified from hospital episodes, which identify live deliveries and stillbirths at greater than 24-weeks’ gestation. To be eligible, women had to (a) have a recorded SMI diagnosis prior to the index pregnancy: schizophrenia, delusional disorder, acute and transient psychosis, schizoaffective disorder, nonorganic psychosis, manic episode, bipolar affective disorder, psychotic depression, or previous puerperal psychosis (ICD-10 F20, F22, F23, F25, F28, F29, F30, F31, F53, or F53.1). To ensure onset of SMI predated pregnancies identified, diagnoses were ascertained at least 9 months before the delivery date; extracted from CRIS either from structured diagnostic fields or through an NLP algorithm and (b) have received contact with SLaM secondary mental health care at any point from 6 months before to 6 weeks after the delivery, so that details of their mental health during the perinatal period would be recorded.

Some women had more than one pregnancy during the study period and for each woman included, we analyzed the first occurring pregnancy ascertained during the sampling period. We wished to analyze incidence of newly occurring severe relapses in the perinatal period, therefore women having a severe relapse at the start of pregnancy were excluded. This was determined by admission to acute psychiatric care (inpatient or home treatment) in the 3 months before the start of pregnancy.

### Exposure—mental health symptom domains

Exposures were mental health symptoms recorded in case notes up to 2 years before pregnancy. NLP algorithms have been developed to extract data on text-recorded symptoms in the electronic mental health record, and they have more recently been supplemented with a range of algorithms for depressive symptoms. The lexicon of symptoms was defined by a team of psychiatrists based on their inclusion in symptom scales common in clinical use, for example the Positive and Negative Symptoms Scale and the Young Mania Rating Scale, incorporating clinical experience of the language used in records, and a priori plans to generate symptom scales based on previous research on the dimensional nature of SMI [29,31,32]. The precision (positive predictive value) and recall (sensitivity) metrics of each modeled symptom for individuals with SMI diagnoses are available open-access [26]. For this study, we extracted 48 symptoms, recorded during the 2 years before the pregnancy start date and grouped symptoms into six domains: positive, negative, disorganization, manic, catatonic, and depressive, allowing some symptoms relevant to more than one domain to be scored in both (e.g., anhedonia as a negative symptom of schizophrenia and a depressive symptom). The domains identified followed the nomenclature used in the research scales used as a source for the lexicon. Positive and negative symptoms conventionally refer specifically to schizophrenia, although were evaluated across the range of SMI. Positive psychotic symptoms included hallucinations and delusions as well as aggression, agitation, and hostility, while negative symptoms encompass those characteristic of decrease or loss of mental function. A full list of symptoms in each domain is provided in Table 1.

**Table 1.** Grouping of symptom-ascertaining natural language processing algorithms into categories.

Symptom profiles	Symptoms included
Positive	Loss of abstract thinking, aggression, agitation, delusion, hallucination, hostility, paranoia, and persecutory ideation
Negative symptoms	Anergia, anhedonia, blunted/flat affect, poor motivation, poverty of speech, poverty of thought, and social withdrawal
Disorganization symptoms	Circumstantial speech, loss of coherence, derailment of speech, flight of ideas, formal thought disorder, and tangential speech
Manic symptoms	Disturbed sleep, elation, elevated mood, grandiosity, insomnia, irritability, and pressured speech
Catatonia symptoms	Catatonic syndrome, echolalia, echopraxia, immobility, mannerism, mutism, perseverance, posturing, stereotype, stupor, and waxy flexibility
Depression symptoms	Anergia, anhedonia, decreased appetite, blunted/flat affect, poor concentration, disturbed sleep, guilt, helpless ideation, hopeless ideation, insomnia, low mood, poor motivation, psychomotor retardation, and worthless ideation

### Outcome—relapse during pregnancy or postpartum

Relapse was defined as an admission to acute mental healthcare, which did not rely on diagnostic information. Acute care was defined as either an inpatient ward or home treatment. Home treatment teams in the UK provide care at home by staff available every day, who can visit up to three times per day, as an alternative to hospital admission [33]. The timing of relapse was recorded as the first date of an inpatient admission or referral to home treatment, and ascertained as to whether it occurred during pregnancy and/ or postpartum—within the first 3 months after the delivery date. Time periods for relapse were not mutually exclusive, such that a relapse could be recorded in both the pregnancy and postpartum periods. Data were extracted from CRIS, supplemented by HES to ascertain psychiatric admissions occurring outside the SLaM catchment area.

### Covariates

Covariates used were selected from relevant literature and data already extracted on the same cohort and previously described [7,8,30]. Data on recorded ethnicity and age at delivery were extracted via CRIS from structured fields, ethnicity was condensed into three categories: “Black African and other Black,” “White British and other White,” and “Asian/Mixed/Other.” Manual text searches were used to establish primiparity, smoking, and relationship status in pregnancy. If a partner or husband was referred to during pregnancy or immediately after birth, “partner in pregnancy” was assigned. No partner was assigned if a woman was single at time of birth or a relationship broke down in pregnancy. Manual searches were also used to ascertain recorded family history of psychosis (schizophrenia, bipolar disorder, or psychotic depression). As patients have varying volumes of clinical notes, the number of clinical documents in the 2 years before pregnancy were also extracted from CRIS.

### Analysis

Statistical analyses were conducted using Stata, version 12 (SataCorp 2011). Descriptive data were generated for baseline socio-demographic and clinical characteristics and for symptoms recorded in the 2 years before the index pregnancy. We counted the number of symptoms within a domain that were identified, and categorized domains into three or four equal groups to determine the distribution of outcomes by symptom numbers in each domain, there being no a priori reason to assume a linear relationship. Associations were then investigated of categorized symptom domains and socio-demographic characteristics with relapse, separately for pregnancy and postpartum periods, using chi-squared tests for categorical variables and *t*-tests for continuous data (e.g., age). Subsequent logistic regression models were assembled with categorized symptom domains entered as ordinal independent variables. Given that women had varying completeness of clinical notes for reasons that could not be clearly determined, we conducted sensitivity analyses to account for potential bias due to missing information by excluding patients with no recorded symptoms. Regression models were first adjusted for covariates chosen a priori from the literature [5,7,16,17,34]. These were age, ethnicity, primiparity, family history of psychosis, smoking, and relationship status in pregnancy. To account for confounding due to volume of clinical information, additional analyses adjusted for number of documents, entered as a categorical variable as there was no a priori reason to assume a linear relationship. To account for correlation of symptom domains confounding each other’s effects, another supplementary analysis further adjusted for all symptom domains.

## Results

### Sample characteristics

The final cohort contained 399 women with SMI with index pregnancies during the study period 2007–2011. Considering diagnoses at the beginning of pregnancy: 145 (36.3%) had a diagnosis of bipolar affective disorder or mania, 28.1% had a diagnosis of schizophrenia, 11.8% acute and transient psychosis, 11.0% psychotic depression, 6.5% schizoaffective disorder, 4.5% psychosis not otherwise specified, and 1.8% had a history of puerperal psychosis only. Considering relapse, 76 (19.0%) of women experienced a relapse during pregnancy and 107 (27.0%) in the first 3 months postpartum. The most common ethnicity was “Black African and Other Black” (47.6%). Mean (standard deviation [SD]) maternal age at delivery was 31.7 (6.0) years, and 67.9% had a partner in pregnancy. Family history of psychosis was present in 14.0% of women and smoking during pregnancy in 18.8%. Just under half of women were primiparous (47.6%) and 23.3% had no text documents in the 2 years before pregnancy, 27.1% had 1–42, 24.8% had 43–127, and 24.8% had 129–1131.

### Symptom domains

Women could be recorded as having symptoms in all or any number of symptom domains. Overall, 115 women had no symptoms recorded in any of the domains in the 2 years before pregnancy. The most common symptoms were: manic 279 (69.9%), followed by depressive 270 (67.7%), positive 247 (62.0%), disorganization 182 (45.6%), negative 145 (36.3%), and catatonic 58 (14.5%) symptoms were the least common. Table 2 shows the distribution of women with symptoms in each categorized symptom domain and summarizes unadjusted associations between symptom domains and relapse. For relapse in pregnancy, chi-squared analyses showed significant categorical associations with positive, disorganization, manic, and catatonic symptoms. For relapse postpartum, significant categorical associations were found with positive, disorganization, manic, and depressive symptoms. Both outcomes appeared to exhibit nonlinear (U-shaped) trends across several of the symptom domains and tests for linear trend were only significant for during-pregnancy relapses with positive and disorganization symptoms.

**Table 2.** Association between symptom domains and relapse during pregnancy and postpartum.

Symptom domains	Total	Pregnancy		Postpartum	
	*N* (%)	Relapse (%)	*χ*^2^ (d.f.); *p*	Relapse (%)	*χ*^2^ (d.f.); *p*
Positive			**12.08 (3); 0.007**		**10.55 (3); 0.014**
0	152 (38.1)	24 (15.8)		48 (31.6)	
1–2	74 (18.6)	8 (10.8)		11 (14.9)	
3–4	73 (18.3)	14 (19.2)		15 (20.6)	
5–8	100 (25.1)	30 (30.0)		33 (33.0)	
*Test for trend*			***7.99 (1); 0.005***		*0.00 (1); 0.959*
Negative			0.87 (2); 0.647		1.93 (2); 0.381
0	254 (63.7)	47 (18.5)		74 (29.1)	
1	85 (21.3)	19 (22.4)		19 (22.4)	
2–5	60 (15.1)	10 (16.7)		14 (23.3)	
*Test for trend*			*0.00 (1); 0.994*		*1.47 (1); 0.225*
Disorganization			**11.30 (2); 0.003**		**7.24 (2); 0.027**
0	217 (54.4)	30 (13.8)		58 (26.7)	
1	93 (23.3)	19 (20.4)		17 (18.3)	
2–5	89 (22.3)	27 (30.3)		32 (36.0)	
*Test for trend*			***11.16 (1); 0.001***		*1.33 (1); 0.249*
Manic			**13.50 (3); 0.004**		**10.02 (3); 0.018**
0	120 (30.1)	26 (18.1)		46 (31.9)	
1–3	85 (21.3)	11 (10.4)		19 (17.9)	
4–6	134 (33.6)	19 (21.4)		20 (22.5)	
6–7	200 (15.0)	20 (33.3)		22 (36.7)	
*Test for trend*			*5.95 (1); 0.015*		*0.00 (1); 0.952*
Catatonic			**4.64 (1); 0.031**		1.22 (1); 0.269
0	341 (85.5)	59 (17.3)		88 (25.8)	
1	58 (14.5)	17 (29.3)		19 (32.8)	
Depressive			1.07 (3); 0.785		**8.47 (3); 0.037**
0	42 (32.3)	22 (17.1)		46 (35.7)	
1–3	112 (28.1)	20 (17.9)		22 (19.6)	
4–6	103 (25.8)	22 (21.4)		25 (24.3)	
7–12	55 (13.8)	12 (21.8)		14 (25.5)	
*Test for trend*			*0.95 (1); 0.329*		*2.82 (1); 0.093*

Abbreviation: d.f., degrees of freedom.

Bold value denote those where p < 0.05 or regression estimates where 95% CI crosses 1.

### Predictors of relapse

Considering covariates, Table 3 displays sample demographic and clinical characteristics according to relapse in pregnancy and postpartum. Relapse during pregnancy, but not postpartum, differed significantly by ethnicity, while both were substantially more common in current smokers. Relapse in pregnancy was also associated with younger age (mean, SD: 30.1 [6.0] vs. 32.1 [6.0] years, *t* = 2.6, *p* = 0.010) but not relapse postpartum (mean [SD]: 31.5 [6.0] and 31.8 [6.0] years, respectively, *t* = 0.4, *p* = 0.697).

**Table 3.** Associations between socio-demographic characteristics and relapse during pregnancy and postpartum (*n* = 399 women).

Characteristic	Pregnancy		Postpartum	
	Relapse *N* (%)	*χ*^2^ (d.f.); *p*	Relapse *N* (%)	*χ*^2^ (d.f.); *p*
Ethnicity		**15.5 (2); <0.001**		2.18 (2); 0.336
White British & other White	12 (8.9)		34 (25.2)	
Black African and other Black	50 (26.3)		57 (30.0)	
Asian/mixed/other	14 (18.9)		16 (21.6)	
Relationship status in pregnancy		2.36 (1); 0.125		0.10 (1); 0.728
Partner	46 (17.0)		74 (27.3)	
No partner	29 (23.4)		32 (25.8)	
Primiparity		0.95 (1); 0.331		0.84 (1); 0.360
Yes	40 (21.1)		55 (29.0)	
No	19 (17.2)		27 (24.9)	
Smoking in pregnancy		**26.30 (1); .001**		**8.18 (1); 0.004**
Yes	30 (40.0)		30 (40.0)	
No	46 (14.2)		77 (23.8)	
Family history of psychosis		0.24 (1); 0.625		0.43 (1); 0.510
Yes	12 (21.4)		13 (23.2)	
No	64 (18.7)		94 (27.4)	

Abbreviation: d.f., degrees of freedom.

Bold value denote those where p < 0.05 or regression estimates where 95% CI crosses 1.

### Multivariable analyses

Multivariable analyses of symptom domains and relapse in pregnancy are displayed in Table 4, showing change in risk of relapse per unit increase in categorized symptom domain. Considering symptom domains entered as ordinal variables, disorganization symptoms showed a positive association at borderline significance with relapse after adjustment (adjusted odds ratio [aOR] = 1.36; 95% confidence intervals [CI] = 0.99–1.87), while other associations were close to the null. Supplementary analyses adjusting for number of documents showed no associations with symptom profiles, and adjusting for other symptom domains showed an independent association with disorganization symptoms (Table S1). In sensitivity analyses excluding patients without any symptoms recorded in the 2 years before pregnancy, higher numbers of positive (1.50; 1.07–2.11), disorganization (1.84; 1.22–2.76), and manic (1.48; 1.03–2.11) symptoms were associated with relapse during pregnancy after adjusting for covariates. Considering relapse in the postpartum period, multivariable analyses (Table 5) indicated a negative association with number of depressive symptoms in fully adjusted analyses (0.78; 0.62–0.98). Supplementary analyses (Table S2) adjusting for all other symptom domains again showed an independent negative association with number of depressive symptoms. In sensitivity analyses excluding women without recorded symptoms in the 2 years prior to pregnancy, the negative association with depressive symptoms was no longer present and disorganization symptoms were associated with relapse (1.54; 1.08–2.20).

**Table 4.** Multivariable analysis of symptom domains and relapse in pregnancy, with symptom variables entered as ordinal categorical variables, *N* = 399 women, 74 with relapse in pregnancy.

Whole sample	OR (95% CI)	Adjusted OR (95% CI)^a^
Positive symptoms	**1.34 (1.09, 1.65), 0.005**	1.09 (0.87, 1.37), 0.462
Negative symptoms	1.00 (0.71, 1.40), 0.993	0.85 (0.58, 1.23), 0.380
Disorganization symptoms	**1.65 (1.22, 2.21), 0.001**	1.36 (0.99, 1.87), 0.060
Manic symptoms	**1.36 (1.06, 1.67), 0.015**	1.11 (0.86, 1.43), 0.424
Catatonic symptoms	**1.98 (1.05, 3.73), 0.034**	1.24 (0.62, 2.47), 0.546
Depressive symptoms	1.13 (0.89, 1.43), 0.329	0.97 (0.74, 1.26), 0.814
***Excluding women with no symptoms***	*N* = 284, 55 relapses	
Positive symptoms	**1.79 (1.29, 2.47), 0.000**	**1.50 (1.07, 2.11), 0.020**
Negative symptoms	0.97 (0.67 1.42), 0.894	0.94 (0.63, 1.42), 0.777
Disorganization symptoms	**2.05 (1.40, 3.02), 0.000**	**1.84 (1.22, 2.76), 0.003**
Manic symptoms	**1.68 (1.20, 2.35), 0.002**	**1.48 (1.03, 2.11), 0.032**
Catatonic symptoms	**2.05 (1.06, 3.99), 0.034**	1.47 (0.72, 3.03), 0.292
Depressive symptoms	1.23 (0.87, 1.76), 0.246	1.21 (0.82, 1.77), 0.334

Abbreviation: CI, confidence intervals; OR, odds ratio.

a Adjusted for age, ethnicity, primiparity, family history of psychosis, smoking, and partner in pregnancy. Bold value denote those where p < 0.05 or regression estimates where 95% CI crosses 1.

**Table 5.** Multivariable analysis of symptom domains and relapse in postpartum, with symptom variables entered as ordinal categorical variables, *N* = 399 women, 107 with relapse in postpartum.

Whole sample	OR (95% CI)	Adjusted OR (95% CI)^a^
Positive symptoms	1.00 (0.84, 1.21), 0.959	0.93 (0.76, 1.13), 0.445
Negative symptoms	0.82 (0.60, 1.13), 0.226	0.78 (0.57, 1.08), 0.139
Disorganization symptoms	1.17 (0.90, 1.53), 0.249	1.08 (0.81, 1.42), 0.603
Manic symptoms	1.01 (0.82, 1.24), 0.952	0.93 (0.75, 1.16), 0.530
Catatonic symptoms	1.40 (0.77, 2.55), 0.271	1.19 (0.64, 2.23), 0.586
Depressive symptoms	0.83 (0.67, 1.03), 0.094	**0.78 (0.62, 0.98), 0.032**
***Excluding women with no symptoms***	*N* = 284, 67 relapses	
Positive symptoms	**1.38 (1.05, 1.82), 0.023**	1.28 (0.96, 1.71), 0.095
Negative symptoms	0.96 (0.68, 1.36), 0.809	0.98 (0.68, 1.41), 0.911
Disorganization symptoms	**1.66 (1.17, 2.34), 0.004**	**1.54 (1.08, 2.20), 0.016**
Manic symptoms	**1.43 (1.06, 1.94), 0.020**	1.34 (0.98, 1.83), 0.070
Catatonic symptoms	**1.81 (0.96, 3.41), 0.068**	1.59 (0.81, 3.10), 0.177
Depressive symptoms	1.00 (0.72, 1.39), 0.993	1.00 (0.71, 1.40), 0.995

Abbreviation: CI, confidence intervals; OR, odds ratio.

a Adjusted for age, ethnicity, primiparity, family history of psychosis, smoking, and partner in pregnancy. Bold value denote those where p < 0.05 or regression estimates where 95% CI crosses 1.

## Discussion

### Key findings

In a relatively large sample of women with SMI diagnoses drawn from a mental healthcare data resource, we sought to investigate the levels of recorded symptoms across different mental health domains in the 2 years before pregnancy and their associations with relapse in pregnancy and/or postpartum. In adjusted models, we found associations of relapse in pregnancy with higher recorded number of disorganization symptoms and relapse postpartum with lower recorded number of depressive symptoms. However, some associations appeared U-shaped. This was mostly on account of the high proportions who had no recorded symptoms, and sensitivity analyses for subgroups with at least one recorded symptom in a given domain enabled examination of the remaining monotonic linear association. Higher numbers of disorganization, positive and manic symptoms were associated with higher risk of relapse during pregnancy, and higher number of disorganization symptoms with higher risk of relapse postpartum.

To the best of our knowledge, this study is unique in its investigation of recorded symptom domains as predictors of perinatal SMI relapses. There has been little research into risk factors for SMI relapse in pregnancy, but associations with higher severity of pre-existing psychiatric illness have been reported including number and recency of previous admissions [3,5,14], and higher risk in non-affective disorders [7]. Previous investigations of hospitalizations in population administrative data have yielded similar or slightly lower estimates than our clinical sample [3,35,36]. The finding of disorganization symptoms being associated with relapse, particularly during pregnancy, is interesting. Disorganization symptoms are found in both nonaffective and affective psychoses and might be a construct common to severe mental disorders. Multivariable sensitivity analyses additionally showed an association between higher numbers of recorded prepregnancy positive or manic symptoms and relapse in pregnancy. While neither was definitively associated with postpartum relapse, both associations fell only marginally outside conventional levels of statistical significance, so negative findings should be viewed with caution.

The association between lower prepregnancy recorded depressive symptoms and postpartum relapse might reflect that women with these symptoms are less likely to require hospitalization and more likely to be managed in the community. Lower risk of perinatal relapses in major depression than bipolar disorder have also been previously reported [1,3]. Negative symptoms in SMI overall predict adverse outcomes such as hospitalization [23], whereas we found no such association with perinatal relapse. However, negative symptoms (e.g., apathy, social withdrawal, and emotional deficits), might render pregnancy less likely, so that those who became pregnant in the presence of this symptomatology might have other protective factors present.

Our findings of more apparent associations with relapse in pregnancy than relapse postpartum require replication; however, although treated as independent outcomes (i.e., a relapse in pregnancy did not exclude the recording of one postpartum), it is possible that at least some of those most vulnerable to relapse experienced this earlier in the perinatal period and either received interventions that prevented postpartum relapse, or else continued to be unwell (or in rehabilitative phases of management) by the time of the index delivery.

In interpreting associations with symptom domains, a noticeable feature was U-shaped associations between several domains and relapse, with higher relapse prevalence in cases with no recorded symptoms. Some of these women may have been inactive patients or a stable group who happened to relapse during the perinatal period. However, it was expected that this might generate differences for all symptom domains, which was not seen. Someone with recorded symptoms might have a more involved clinical team providing higher levels of clinical care in other respects. Conversely, women with no symptoms recorded might have a less engaged team who were not identifying or recording pathology. These effects might have thus diluted or complicated associations. A further explanation might be that women with more severe illnesses may have symptoms on all the domains, as adjusting for the other symptom domains did attenuate the findings.

### Strengths and limitations

One of the key strengths of this study was the size and generalizability of our sample to patients in secondary mental health care. Using clinical records allowed a larger and more inclusive sample, such as very unwell or otherwise marginalized women than would be likely to be achieved from *de novo* recruitment. For associations of interest, statistical power was likely adequate and upper confidence intervals for null associations do not suggest strong underlying associations were missed. However, some exposures were relatively rare (e.g., catatonic symptoms) and need further assessment in larger samples. The SLaM catchment has relatively high ethnic and social diversity, and dynamic migration patterns [25], and may not generalize to rural areas. In relation to SMI, only women managed by secondary care will have been included, thus missing cases managed exclusively in primary care. Considering generalizability in relation to pregnancy, HES data do not include home births, although these accounted for only 2.4% of births in England in 2011; furthermore, the likelihood of home deliveries in women with SMI can be expected to be lower, as pregnancies are usually considered high-risk obstetrically [37].

Limitations naturally arise from the use of routine clinical data; information is collected for clinical and administrative purposes and may be missing or more varying in accuracy than that collected for research. For example, a number of covariates relied on free text. If information is missing (such as for smoking or family history) it is difficult to ascertain if it is absent or just not recorded, resulting in potential misclassification. Furthermore, more unwell women including those with severe relapses likely have more clinical information recorded, which could lead to residual confounding, although attempts were made to correct for this by conducting sensitivity analyses excluding women with no recorded symptoms, and through adjustment for document numbers.

Considering relapse ascertainment, the linkage with HES and its full coverage of England, allowed follow-up for relapses in women who moved away from the area. However, admission to acute care may not be a very sensitive indicator of relapse, as minor relapses can be managed by other secondary services. In this respect, home treatment team referral was only ascertained from local (SLaM) records. Considering the exposures of interest, symptoms are subjective measures based on a healthcare professional’s interpretation, and the performance of the NLP algorithm. In addition, we imposed an a priori categorization approach to symptoms in this study, albeit using domains commonly applied in other fields and derived from established scales and clinician opinion, rather than generated empirically from clustering in the data. Negative symptoms are difficult to assess and may be less documented than the more clinically evident positive symptoms. In general, suboptimal performance of the NLP algorithms for ascertaining symptoms will have resulted in measurement error and obscured rather than exaggerated outcome associations of interest. Finally, while chosen as a pragmatic approach for this initial investigation, simply counting the number of symptoms within a given domain may miss complexity, for example, the severity, pervasiveness, and occurrences of a given symptom and the potential interaction between symptoms within a given domain.

### Implications and conclusions

Considering future research and clinical application, the use of routine healthcare data for cohorts who are difficult to recruit in large numbers has potential for identifying predictors of relapse, particularly with information extraction techniques. In our setting, linked hospitalization data was key to identifying pregnancies. In light of the study’s findings, further research in larger cohorts may yield higher precision estimates of the associations of interest as well as assessing replicability. The applicability of symptom domains as predictors of relapse needs further clarification before interventions can be considered. However, there are at least some indications that symptomatic risk factors cross diagnostic groupings and further work might seek to evaluate empirically whether these are more accurate than diagnoses for identifying risk of relapse.

## Data Availability

The data that support the findings of this study are available from the authors, subject to permission from the South London and Maudsley NHS Foundation Trust and appropriate procedures for access within the Trust’s network in accordance with current approvals.

## Supplementary material

For supplementary material accompanying this paper visit http://dx.doi.org/10.1192/j.eurpsy.2021.18. click here to view supplementary material
