# Supplementary material for: An evaluation of symptom domains in the 2 years before pregnancy as predictors of relapse in the perinatal period in women with severe mental illness
Source: Eur Psychiatry. 2021 Mar 19;64(1):e26. doi: 10.1192/j.eurpsy.2021.18 (PMC8082469; doi:10.1192/j.eurpsy.2021.18)
Supplement: Supplementary file 1 [file epasup.zip › S0924933821000183sup002.docx]

*Table S2: Multivariable analysis of symptom domains and relapse in postpartum, with symptom variables entered as ordinal categorical variables, N=399 women, 107 with relapse in postpartum*

|  | **OR (95% CI)** | **adjusted model 1:**  **OR (95% CI)** | **adjusted model 2:**  **OR (95% CI)** | **adjusted model 3:**  **OR (95% CI)** |
| --- | --- | --- | --- | --- |
| ***Whole sample*** |  |  |  |  |
| Positive symptoms | 1.00 (0.84, 1.21), 0.959 | 0.93 (0.76, 1.13), 0.445 | 1.08 (0. 79, 1.48), 0.635 | 0.98 (0.68, 1.41), 0.921 |
| Negative symptoms | 0.82 (0.60, 1.13), 0.226 | 0.78 (0.57, 1.08), 0.139 | 0.84 (0.57, 1.24), 0.383 | 1.02 (0.62, 1.68), 0.942 |
| Disorganisation symptoms | 1.17 (0.90, 1.53), 0.249 | 1.08 (0.81, 1.42), 0.603 | 1.45 (0.99, 2.13), 0.057 | 1.48 (0.96, 2.27), 0.076 |
| Manic symptoms | 1.01 (0.82, 1.24), 0.952 | 0.93 (0.75, 1.16), 0.530 | 1.14 (0.80, 1.62), 0.462 | 1.29 (0.83, 2.01), 0.257 |
| Catatonic symptoms | 1.40 (0.77, 2.55), 0.271 | 1.19 (0.64, 2.23), 0.586 | 1.47 (0.74, 2.90), 0.268 | 1.47 (0.71, 3.02), 0.297 |
| Depressive symptoms | 0.83 (0.67, 1.03), 0.094 | **0.78 (0.62, 0.98), 0.032** | 0.73 (0.51, 1.04), 0.082 | **0.57 (0.35, 0.94), 0.029** |
| ***Excluding women with no recorded symptoms (N=284, 67 with relapses)*** | | |  |  |
| Positive symptoms | **1.38 (1.05, 1.82), 0.023** | 1.28 (0.96, 1.71), 0.095 | - | 1.08 (0.74, 1.57), 0.695 |
| Negative symptoms | 0.96 (0.68, 1.36), 0.809 | 0.98 (0.68, 1.41), 0.911 | - | 0.88 (0.52, 1.51), 0.646 |
| Disorganisation symptoms | **1.66 (1.17, 2.34), 0.004** | **1.54 (1.08, 2.20), 0.016** | - | 1.43 (0.92, 2.22), 0.114 |
| Manic symptoms | **1.43 (1.06, 1.94), 0.020** | 1.34 (0.98, 1.83), 0.070 | - | 1.28 (0.82, 2.00), 0.276 |
| Catatonic symptoms | **1.81 (0.96, 3.41), 0.068** | 1.59 (0.81, 3.10), 0.177 | - | 1.43 (0.69, 2.96), 0.335 |
| Depressive symptoms | 1.00 (0.72, 1.39), 0.993 | 1.00 (0.71, 1.40), 0.995 | - | 0.74 (0.42, 1.29), 0.287 |

**Adjusted Model 1: adjusted for age, ethnicity, primiparity, family history of psychosis, smoking and partner in pregnancy**

**Adjusted Model 2: adjusted for age, ethnicity, primiparity, family history of psychosis, smoking, partner in pregnancy and number of documents**

**Adjusted Model 3: adjusted for age, ethnicity, primiparity, family history of psychosis, smoking, partner in pregnancy, and all symptom profile categories**
