# Supplementary material for: An evaluation of symptom domains in the 2 years before pregnancy as predictors of relapse in the perinatal period in women with severe mental illness
Source: Eur Psychiatry. 2021 Mar 19;64(1):e26. doi: 10.1192/j.eurpsy.2021.18 (PMC8082469; doi:10.1192/j.eurpsy.2021.18)
Supplement: Supplementary file 1 [file epasup.zip › S0924933821000183sup001.docx]

*Table S1: Multivariable analysis of symptom domains and relapse in pregnancy, with symptom variables entered as ordinal categorical variables, N=399 women, 74 with relapse in pregnancy*

|  | **OR (95% CI)** | **adjusted model 1:**  **OR (95% CI)** | **adjusted model 2:**  **OR (95% CI)** | **adjusted model 3:**  **OR (95% CI)** |
| --- | --- | --- | --- | --- |
| ***Whole sample*** |  |  |  |  |
| Positive symptoms | **1.34 (1.09, 1.65), 0.005** | 1.09 (0.87, 1.37), 0.462 | 1.04 (0.72, 1.51), 0.830 | 0.96 (0.63, 1.46), 0.833 |
| Negative symptoms | 1.00 (0.71, 1.40), 0.993 | 0.85 (0.58, 1.23), 0.380 | 0.70 (0.44, 1.09), 0.116 | 0.75 (0.42, 1.31), 0.311 |
| Disorganisation symptoms | **1.65 (1.22, 2.21), 0.001** | 1.36 (0.99, 1.87), 0.060 | 1.50 (0.97, 2.33), 0.069 | **1.61, (0.98, 2.64), 0.058** |
| Manic symptoms | **1.36 (1.06, 1.67), 0.015** | 1.11 (0.86, 1.43), 0.424 | 1.07 (0.71, 1.61), 0.749 | 1.06 (0.65, 1.75), 0.808 |
| Catatonic symptoms | **1.98 (1.05, 3.73), 0.034** | 1.24 (0.62, 2.47), 0.546 | 1.14 (0.55, 2.39), 0.722 | 1.20 (0.55, 2.61), 0.643 |
| Depressive symptoms | 1.13 (0.89, 1.43), 0.329 | 0.97 (0.74, 1.26), 0.814 | 0.77 (0.51, 1.16), 0.219 | 0.78 (0.44, 1.37), 0.558 |
| ***Excluding women with no recorded symptoms (N=284, 55 with relapses)*** | | |  |  |
| Positive symptoms | **1.79 (1.29, 2.47), 0.000** | **1.50 (1.07, 2.11), 0.020** | - | 1.26 (0.81, 1.95), 0.310 |
| Negative symptoms | 0.97 (0.67 1.42), 0.894 | 0.94 (0.63, 1.42), 0.777 | - | 0.61 (0.34, 1.12), 0.112 |
| Disorganisation symptoms | **2.05 (1.40, 3.02), 0.000** | **1.84 (1.22, 2.76), 0.003** | - | 1.57 (0.94, 2.62), 0.085 |
| Manic symptoms | **1.68 (1.20, 2.35), 0.002** | **1.48 (1.03, 2.11), 0.032** | - | 1.13 (0.68, 1.87), 0.628 |
| Catatonic symptoms | **2.05 (1.06, 3.99), 0.034** | 1.47 (0.72, 3.03), 0.292 | - | 1.24 (0.56, 2.71), 0.594 |
| Depressive symptoms | 1.23 (0.87, 1.76), 0.246 | 1.21 (0.82, 1.77), 0.334 | - | 1.12 (0.60, 2.11), 0.714 |

**Adjusted Model 1: adjusted for age, ethnicity, primiparity, family history of psychosis, smoking and partner in pregnancy**

**Model 2 = adjusted for age, ethnicity, primiparity, family history of psychosis, smoking, partner in pregnancy and number of documents**

**Adjusted Model 3 = adjusted for age, ethnicity, primiparity, family history of psychosis, smoking, partner in pregnancy, and all symptom profile categories**
